# Mendelian Randomization Reveals Genetic Associations Between Immune Traits and Urethral Stricture

**DOI:** 10.1155/mi/3748167

**Published:** 2026-02-27

**Authors:** Qiang Guo, Jin-Cheng Guo, Zhan-Long Zheng, Ke Yang, Jing-Qi Wang, Cheng-Yong Li, Yu-Jia Xi

**Affiliations:** ^1^ Department of Urology, Second Hospital of Shanxi Medical University, 382 Wuyi Road, Taiyuan, 030001, China, sxmu.edu.cn; ^2^ Male Reproductive Health Research Center, Shanxi Medical University, 55 Wenhua Street, Jinzhong, 030600, China, sxmu.edu.cn

**Keywords:** causal association, fibrosis, immune cells, Mendelian randomization, urethral stricture

## Abstract

**Background:**

There is currently little research on the association between immune traits and urethral stricture, and the diversity of immune cell subtypes leads to an unclear association with urethral stricture. This study investigated the relationship between immune cells and urethral stricture using the Mendelian randomization (MR) technique.

**Methods:**

We assessed the causative relationships between urethral stricture (*N* = 341,285) and 731 immunological characteristics (*N* = 3757). The main technique was the inverse‐variance weighted (IVW) approach, which was supplemented by MR‐Egger and weighted median (WM). The robustness of MR results was assessed using sensitivity analyses, primarily the Egger intercept, Cochran’s *Q* test, MR‐PRESSO, and leave‐one‐out test.

**Results:**

Our findings revealed that 37 immune phenotypes have suggestive causal relationships with urethral stricture, including 13 subtypes of B cells, four classical dendritic cells, three myeloid cells, five monocytes, four TBNK, five Treg cells, and three maturation stages of T cells. Sensitivity analyses revealed neither heterogeneity nor pleiotropy.

**Conclusions:**

Our findings supported a potential causal relationship between immune cells and urethral stricture. This may guide the management and treatment of urethral stricture in the future.

## 1. Introduction

The constriction of the urethra, primarily due to scarring, is known as urethral stricture [1, 2]. It can lead to difficulty urinating, accompanied by infections, obstruction of the urethra, and even damage to the function of the entire urinary system, causing great harm to patients, especially males [3, 4]. The incidence rate of urethral stricture is 0.6%−0.9% in developed countries, while it is higher in developing countries. The urethral stricture is a heavy economic burden, estimated to cause $200 million in losses annually in the United States [5].

Previous research has revealed that urethral stricture is mainly caused by fibrosis caused by urethral infections or injuries. Researchers have recently focused more on the part immune cells play in the development of fibrosis [6]. There is evidence that CD4 T + H17 cells contribute to inflammation and fibrosis by secreting proinflammatory cytokines such as IL‐17A by the IL‐1β‐IL‐17A‐TGF‐β1 cytokine axis [7, 8]. It has also been reported that type 2 cytokine response contributes significantly to progressive fibrosis [9, 10]. A study conducted by Ekerhult et al. [11] showed that ectopic germinal centers of B cells, T cells, and follicular dendritic cell networks in the urethral stricture tissues. In the above studies, urethral stricture was found to be associate with immune cells, but a causal relationship has not been established.

Using single nucleotide polymorphisms (SNPs) as instrumental variables (IVs), the Mendelian randomization (MR) approach is a revolutionary epidemiological analytic technique that determines causal links between exposures and outcomes [12, 13]. It is true that causal relationships can only be established through randomized controlled trials; however, they also have limitations, including ethical issues, uncontrollable external factors, costs, and sensitivity to confounding factors [14]. The MR method effectively avoids these problems [15].

This study used publicly accessible GWAS data to conduct a two‐sample MR analysis in order to ascertain whether there might be a causal association between the urethral stricture and 731 immune cells.

## 2. Materials and Methods

Study summary datasets were obtained from publicly available studies approved by the institution itself in their respective studies. No additional approvals were required, as every piece of data used was already publicly available. To guarantee the integrity of our procedures in Supporting Information 1: S1 Appendix, we have included the STROBE‐MR checklist of MR investigations based on earlier research [16, 17].

### 2.1. Study Overview

**Figure 1:**
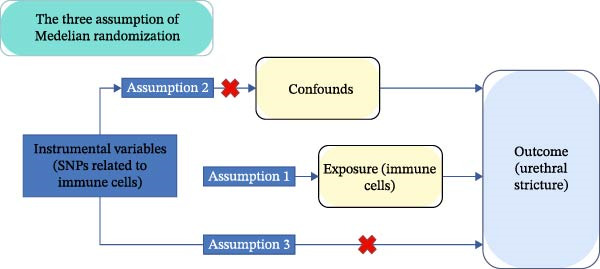
Graphic representation of MR and its three main tenets. Genetic variants should be substantially linked to immunophenotype, according to the first assumption; genetic variants retrieved for immunophenotype should not be influenced by any confounders linked to both immunophenotype and urethral stricture, according to the second assumption. According to the third premise, exposure is the sole way that genetic variations impact urethral stricture.

Two‐sample MR analyses were used to evaluate the causal link between immune cells and urethral stricture. MR studies should follow three major assumptions: (i) the correlation hypothesis: IVs selected are directly related to immune cells; (ii) the independence hypothesis: the selected IVs are unrelated to any factors that could confuse the immune cells and urethral stricture; (iii) the exclusion restriction hypothesis: the selected IV does not affect urethral stricture unless they affect the immune cells [18](Figure 1).

### 2.2. GWAS Summary Statistics of Immune Cells

The most recent immune cell GWAS summary statistics (the archive numbers = GCST0001391–GCST0002121) were used (https://opengwas.io/datasets/) (Supporting Information 2: S2 Appendix). After controlling for confounders including sex, age, and year, almost 240,000 SNPs were genotyped in this study, including 731 different immune phenotypes based on a reference panel with Sardinian sequences [19].

### 2.3. GWAS Summary Statistics of the Urethral Stricture

The latest GWAS data for the urethral stricture from the FinnGen Consortium (https://www.finngen.fi/en) was used, which study contains 1711 cases and 339,574 controls [20]. It was accomplished by the national Finnish biobank network and aims to collect and analyze genetic information from over 500,000 participants. One of its strengths is that it uses the entire population of Finland, which can avoid sample overlap. The other strength is the university‐hospital‐based recruitment, which captured more cases and distinguished them from many working‐age population cohorts (Supporting Information 3: S3 Appendix).

### 2.4. Selection of IVs

To get appropriate SNPs, a rigorous quality control approach was implemented. The steps were as follows: (1) First, even though the significance criterion is often set at *p*  < 5 × 10 − 8, in this investigation, sufficient SNPs were not found as IVs, so we relaxed the restriction and chose *p*  < 1 × 10 − 5 as the significance threshold, which was implemented in previous studies [21]. (2) The independence of the selected SNPs was evaluated using pairwise‐linkage disequilibrium, and SNPs in linkage disequilibrium were eliminated (*r* ^2^ >0.001 and clumping window <10,000 kb). (3) The *F*‐statistic was computed using the formula $F=\frac{R^{2}\left(n-k-1\right)}{\left(1-R^{2}\right)k}$ in order to evaluate the strength of the chosen SNPs, where *R* ^2^ is the contribution to variability of each SNP, *N* is the GWAS sample size, and *k* is the number of SNPs. An *F* < 10 was considered to be a suspected bias [22]. To increase robustness, we used a phenotype analysis web (http://www.phenoscanner.medschl.cam.ac.uk) to analyze the relationship between immune cell SNPs that have been published and other confounding variables, which can also verify the applicability of the IVs [23]. We uploaded the SNPs found in the previous steps on the website, checking if they are related to the risk factors we have identified (lichen sclerosis, infectious urethritis, and circumcision), and attempted to exclude SNPs related to these risk factors.

### 2.5. Statistical Analyses

To make sure the effect sizes for each GWAS were correlated with the same alleles, data from immune cells and urethral stricture for all IVs were harmonized. In this work, causality was evaluated using the conventional inverse‐variance weighted (IVW) assessment method. Estimates from this approach have been found to be fairly accurate when directed pleiotropy and immune cell and urethral stricture heterogeneity are absent [24]. Additionally, the MR‐Egger and weighted median (WM) approaches were employed to guarantee the analysis’s robustness [25].

The MR estimates were then tested and corrected for robustness using sensitivity analyses. First, the MR‐Egger intercept was used to evaluate the SNP diversity [26]. A low pleiotropy was thought to exist when the intercept was near zero. Next, we evaluated the heterogeneity using Cochran’s *Q* test [27]. To ensure the robustness of the data, a leave‐one‐out test was employed to assess the stability of the MR results by removing IVs one at a time [28]. Finally, funnel plots were conducted to test if the result was affected by potential biases. Additionally, to look for outliers that might be polyvalent, the MR‐PRESSO test was run [29]. In addition, we also conducted reverse MR analysis to verify the possibility of a bidirectional causal relationship between the two [30].

The “TwoSampleMR” R package (version 0.5.10) and the “MRPRESSO” R package were used to perform all MR studies using R software (version 4.3.1). The R code used has been summarized in Supporting Information 4: S4 Appendix.

## 3. Results

### 3.1. SNP Selection

**Figure 2:**
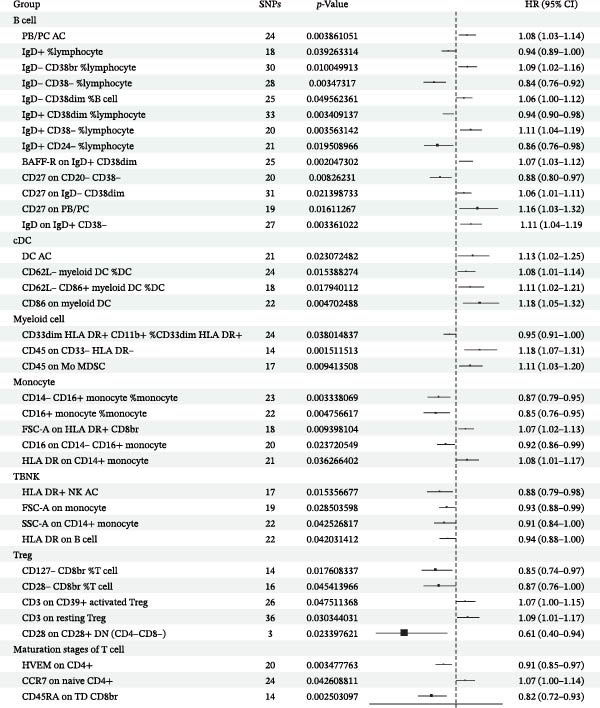
Forest plot of MR results of the association between the immune phenotype of immunophenotype cells and risk of urethral stricture by IVW. CI, confidence interval; IVW, inverse‐variance weighted; MR, Mendelian randomization; OR, odds ratio; SNP, single‐nucleotide polymorphism.

In the study, phenotypes of immune cells were employed as exposure, while urethral stricture was used as an endpoint to look for SNPs as IVs. As mentioned above, all IVs were strongly correlated with exposure (*p*  < 1 × 10 − 5). There are no weak IVs for the *F*‐statistic, for every IV we examined was higher than 10. We found no SNPs in our results associated with other confounding factors. The overall research results can be seen in the Supporting Information 5: S5 Appendix, and all the results of the 37 immune cell morphologies were summarized in Figure 2.

Following MR statistical analysis, we discovered that 37 immune cell morphologies out of 731 were causally linked to urethral stricture (*p*  < 0.05).

### 3.2. B Cell

Using the IVW approach, we identified 13 immune phenotypes of B cells that were causally associated with the urethral stricture. They were as follows: PB/PC AC (odds ratio [OR] = 1.08, 95% confidence interval [CI]): 1.03–1.14, *p* = 0.01), IgD+ %lymphocyte (OR = 0.94, 95% CI: 0.89–1.00, *p* = 0.04), IgD− CD38br %lymphocyte (OR = 1.09, 95% CI: 1.12–1.16, *p* = 0.01), IgD− CD38− %lymphocyte (OR = 0.84, 95% CI: 0.76–0.92 *p* = 0.01), IgD− CD38dim %B cell (OR = 1.06, 95% CI: 1.00–1.12 *p* = 0.05), IgD+ CD38dim %lymphocyte (OR = 0.94, 95% CI: 0.90–0.98 *p* = 0.01), IgD+ CD38− %lymphocyte (OR = 1.11, 95% CI: 1.04–1.19 *p* = 0.01), IgD+ CD24− %lymphocyte (OR = 0.86, 95% CI: 0.76–0.98 *p* = 0.02), BAFF‐R on IgD+ CD38dim (OR = 1.07, 95% CI: 1.03–1.12 *p* = 0.01), CD27 on CD20− CD38− CD27 on CD20− CD38− B cell (OR = 0.88, 95% CI: 0.80–0.97 *p* = 0.01), CD27 on IgD− CD38dim (OR = 1.06, 95% CI: 1.01–1.11 *p* = 0.02), CD27 on PB/PC (OR = 1.16, 95% CI: 1.03–1.32 *p* = 0.02), IgD on IgD+ CD38− (OR = 1.11, 95% CI: 1.04–1.19 *p* = 0.01).

### 3.3. Classic Dendritic Cells

For classic dendritic cells, four immune phenotypes were causally associated with urethral stricture, which included dendritic cell absolute count (OR = 1.13, 95% CI: 1.02–1.25 *p* = 0.02), CD62L− myeloid DC %DC (OR = 1.08, 95% CI: 1.01–1.14 *p* = 0.02), CD62L− CD86+ myeloid DC %DC (OR = 1.11, 95% CI: 1.02–1.21 *p* = 0.02) and CD86 on myeloid DC (OR = 1.18, 95% CI: 1.05–1.32 *p* = 0.01).

### 3.4. Myeloid Cell

We found three immune phenotypes about myeloid cell were causally related with urethral stricture, including CD33dim HLA DR + CD11b+ %CD33dim HLA DR+ (OR = 0.95, 95% CI: 0.91–1.00 *p* = 0.04), CD45 on CD33− HLA DR− (OR = 1.18, 95% CI: 1.07–1.31 *p* = 0.01), CD45 on Mo MDSC (OR = 1.11, 95% CI: 1.03–1.20 *p* = 0.01).

### 3.5. TBNK

Four immune phenotypes about TBNK were found to be basally related with urethral stricture, including HLA DR + NK AC (OR = 0.88, 95% CI: 0.79–0.98 *p* = 0.02), FSC‐A on monocyte (OR = 0.93, 95% CI: 0.88–0.99 *p* = 0.03), SSC‐A on CD14+ monocyte (OR = 0.91, 95% CI: 0.84–1.00 *p* = 0.04), HLA DR on B cell (OR = 0.94, 95% CI: 0.88–1.00 *p* = 0.04).

### 3.6. Monocyte

Five immune phenotypes associated with monocyte were considered to have a causal association with urethral stricture, including CD14− CD16+ monocyte %monocyte (OR = 0.87, 95% CI: 0.79–0.95 *p* = 0.01), CD16+ monocyte %monocyte (OR = 0.85, 95% CI: 0.76–0.95 *p* = 0.01), FSC‐A on HLA DR + CD8+ T cell (OR = 1.07, 95% CI: 1.02–1.13 *p* = 0.01), CD16 on CD14− CD16+ monocyte (OR = 0.92, 95% CI: 0.86–0.99 *p* = 0.02), HLA DR on CD14+ monocyte (OR = 1.08, 95% CI: 1.01–1.17 *p* = 0.07).

### 3.7. Treg

Our results revealed that five immune phenotypes associated with Treg have a causal association with urethral stricture, including CD127− CD8br %T cell (OR = 0.85, 95% CI: 0.74–0.97 *p* = 0.02), CD28− CD8br %T cell (OR = 0.87, 95% CI: 0.76–1.00 *p* = 0.05), CD3 on CD39+ activated Treg (OR = 1.07, 95% CI: 1.00–1.15 *p* = 0.05), CD3 on resting Treg (OR = 1.09, 95% CI: 1.01–1.17 *p* = 0.03), CD28 on CD28 + CD4− CD8− T cell (OR = 0.61, 95% CI: 0.40–0.94 *p* = 0.02).

### 3.8. Maturation Stages of T Cell

We found three immune phenotypes about maturation stages of T cells that have a causal association with urethral stricture, such as herpes virus entering the medium (HVEM) on CD4+ T cell (OR = 0.91, 95% CI: 0.81–0.97 *p* = 0.01), CCR7 on naive CD4+ T cell (OR = 1.07, 95% CI: 1.00–1.14 *p* = 0.04), CD45RA on terminally differentiated CD8+ T cell (OR = 0.82, 95% CI: 0.72–0.93 *p* = 0.01).

### 3.9. Sensitivity Analysis

**Figure 3:**
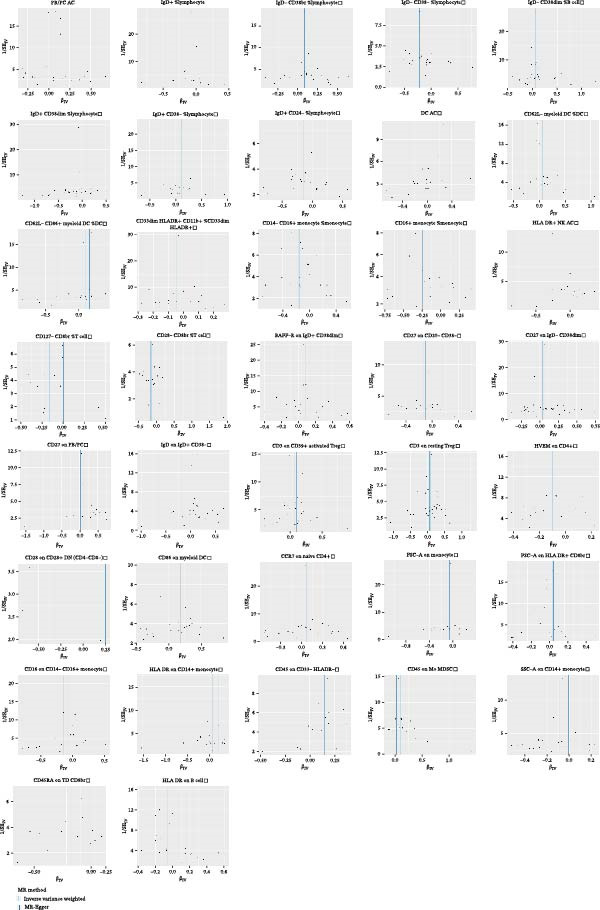
The scatter plots for MR analyze the causal effect between immune cells and urethral stricture using the conventional IVW, MR‐Egger, and weighted median.

**Figure 5:**
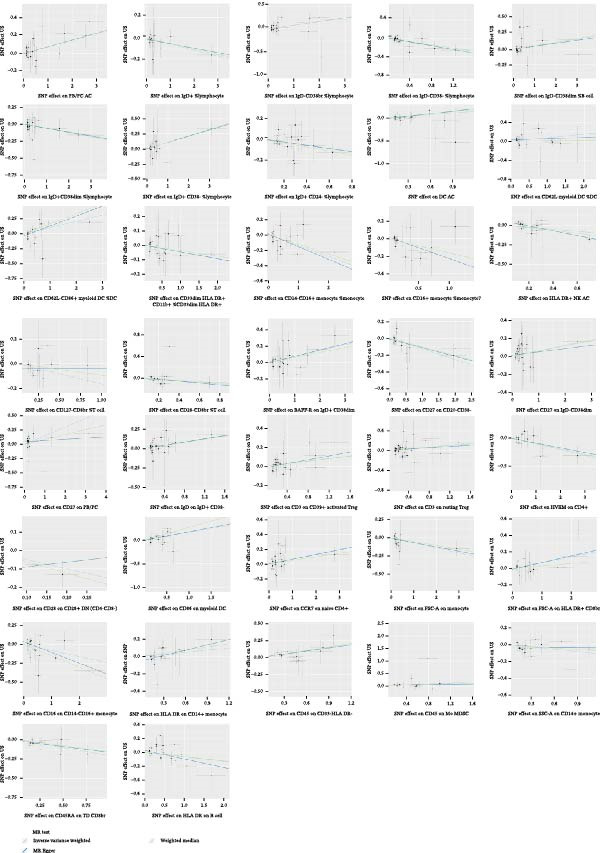
IVW and MR‐Egger regression slopes were used to explore asymmetry as a sign of pleiotropy of the effect between immune cells and urethral stricture, with the vertical line in the middle indicating the sum of different effect sizes.

**Figure 6:**
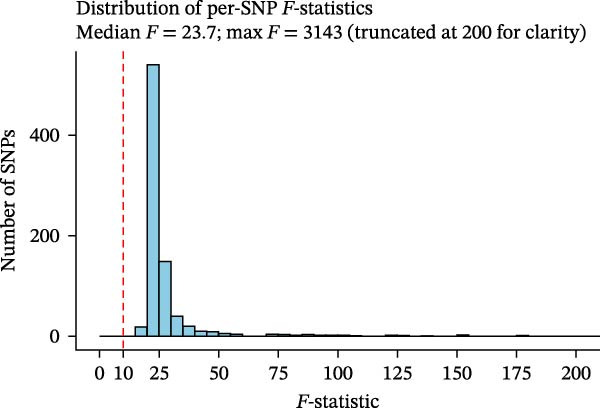
Distribution diagram of the *F*‐statistic. A *F*‐value greater than 10 indicates a higher strength of the effect of the result.

Heterogeneity was measured using Cochran’s *Q* test, which indicated that there was no substantial heterogeneity among SNPs because every set of results had a *p* > 0.05 (Supporting Information 6: S6 Appendix). The funnel plots suggested that the causal relationship was unlikely to be affected by potential biases in the symmetric distribution of SNPs (Figure 3). Additionally, the leave‐one‐out sensitivity analysis revealed no outliers, suggesting that the MR results were not caused by a SNP (Figure 4). The MR‐Egger intercept test revealed directional pleiotropy in two sets of findings (CD27 on PB/PC (intercept of 0.05, *p* = 0.02), HLA DR + NK AC (intercept of 0.05, *p* = 0.03) (Figure 5, Supporting Information 7: S7 Appendix). However, no outliers were found in these two sets of results by the MR‐PRESSO test (CD27 on PB/PC (ID: ebi‐a‐GCST90001807) (Global Test *p* value = 0.56), HLA DR + NK AC (ID: ebi‐a‐GCST90001648) (Global Test *p* value = 0.17) (Supporting Information 8: S8 Appendix), indicating that the results are still reliable [31–33]. In addition, we plotted a distribution diagram of the *F*‐statistic, which suggests that our results have a high effect intensity (*F* < 10) (Figure 6). Besides, the results of reverse MR showed a causal relationship between urethral stricture and 37 out of 731 immune phenotypes, which reveals the possibility of a bidirectional causal relationship between them (Supporting Information 9: S9 Appendix). All these analyses showed that our results were sufficiently robust.

Figure 4Leave‐one‐out analysis was used to determine whether any SNP drove the causal association of immune cells on urethral stricture, which repeated the IVW analysis by discarding each exposure‐related SNP.

**Figure:**
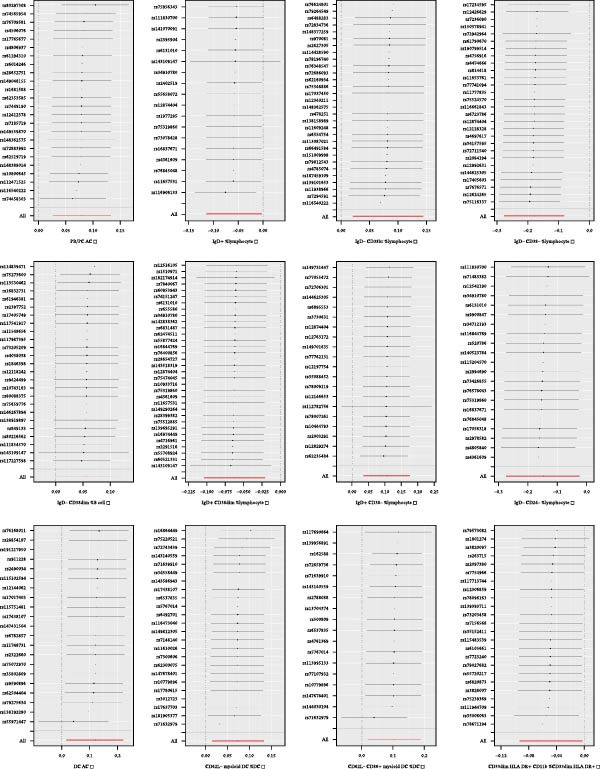

**Figure:**
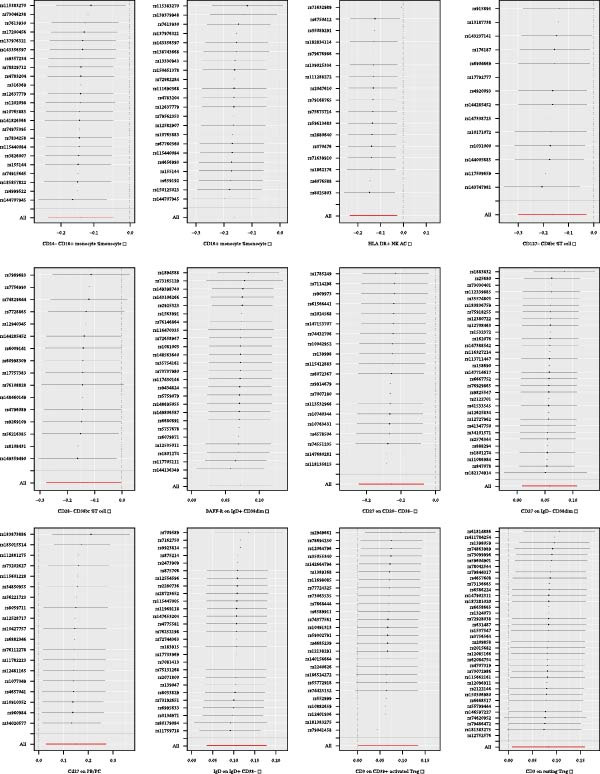

**Figure:**
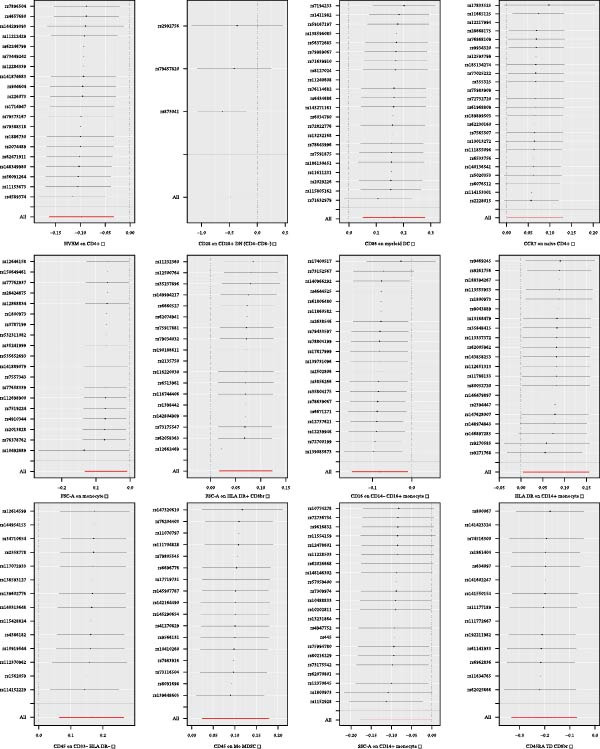

**Figure:**
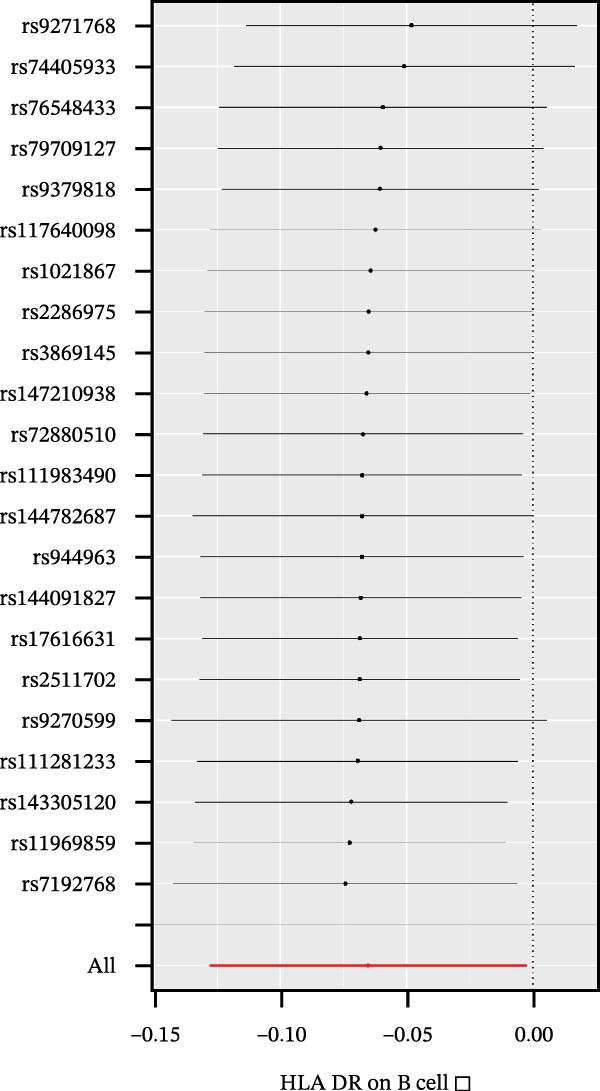

## 4. Discussion

This study used a two‐sample MR technique to evaluate the causal links of immune cells and urethral stricture, and identified 37 immune cell morphologies with a causative relationship to urethral stricture. This is the first MR study that evaluates the role that immune cells play in the process of urethral stricture that we know of. This might illustrate how important immune cells are to the growth and trajectory of the urethral stricture.

Despite the fact that no studies have shown a clear link between immune cells and urethral stricture, some studies have reported that B cells, T cells, and a network of follicular dendritic cells are found in ectopic germinal centers of urethral stricture tissues, which provided a possibility for the inflammatory‐related to immune cells on urethral stricture [11]. Studies have suggested that the effectiveness of urogenital surgery can be enhanced by using human acellular dermal matrix or inhibitors, which can effectively reduce the immunoreaction [34]. According to our findings, there is a direct correlation between immunological phenotype and urethral stricture, supporting the notion of the previously cited study.

It is unclear how immune cells work in urethral stricture. However, urethral stricture, as a disease closely related to the process of fibrosis, is also related to the function of immune cells, especially lymphocytes, closely [35]. Allogeneic immunity stimulates the B cell differentiation into plasma cells by stimulating CD4 T cells with specificity. Additionally, it generates immunologically active molecules like IgD, which has been shown to be important in the progression of fibrosis in other human body parts [36–38]. It has long been known that these cells play a complex function in different types of fibrosis. In the process of fibrosis in other organs, D38+ or CD25+ plasma cells were considered to promote fibrosis, which is consistent with our results [39]. On the contrary, CD8br T cells and HLA DR on B cells are protective factors. These were proven to be protective factors in fibrosis in other parts of the body [40–42]. In addition, lymphocytes also participate in the inflammatory response process of the tissues through various pathways, such as vitamin D antibody‐related pathways, which may also be related to the occurrence and development of urethral stricture [43–45]. Although the mechanism in urethral stricture is still unclear, these immune phenotypes in our results further confirm this.

As for dendritic cells, in our results, their immune phenotype was a risk factor for urethral stricture. Dendritic cells, a type of traditional antigen‐presenting cell, have already been shown to be crucial in the development of inflammation [46]. Likewise, they are critical to the progression of fibrosis in various body parts [47]. In addition, except for antigen presentation, a study revealed that in skin fibrosis, they may produce epidermal growth factors to maintain inflammation and fibrosis, which may explain a part of the promoting role they play in the development of urethral stricture [48].

Prior research has shown that the CD14 + CD16 mononuclear phagocyte system in milder inflammatory areas exerts an impact on local epithelial and mesenchymal cells through the highly enriched ExM and its precursor Ly‐6C on their surface, ultimately promoting scar formation and exacerbating fibrosis progression [49, 50]. The ExM can promote the generation of galactin‐3 and arginase, and it serves as a PAI‐1 source in mice suffering from type II AEC damage mediated by DT. These immune‐active substances all play an important role in fibrosis [51–53]. Meanwhile, the monocyte chemotactic protein 1 produced during the action of the monocyte‐macrophage system may promote DC recruitment and expansion, which is strongly associated with fibrosis [54, 55]. Furthermore, the mononuclear phagocytic system plays an important role in processes such as coagulation, thrombosis, and infection, which may be potentially associated with pathological changes in scar tissue [56, 57]. The above effects are consistent with our findings.

Hepatic stellate cell (HSC) is confirmed to be associated with liver fibrosis [58]. NK performed an anti‐fibrotic property in the liver by inhibiting the activation of HSC populations [2]. By activating HSCs via the TNF‐α‐TNFR1‐NLRP3 signaling axis, DNTs accelerate the advancement of fibrosis [59]. In our results, NK and DNTs have a similar role in urethral fibrosis, and thus, we speculate that these immune cells may have a similar mechanism to liver fibrosis in urethral stricture.

We also found immune phenotypes of IgD, CD45, and FSC‐A‐related presented inconsistent effects, which were generally considered risk factors for fibrosis in previous studies [60–62]. Myeloid cells can reduce fibrosis by inhibiting macrophage function, which was widely recognized in other organs; however, inconsistent with our research [63, 64]. It has been reported in renal fibrosis that Tregs inhibit inflammatory cells by boosting CCL28 production [65]; however, this was inconsistent with our research that revealed Tregs will increase the risk of urethral stricture. This study also considers the HVEM a protective factor of urethral stricture, which is also seen as a risk factor in the fibrosis of other parts [66]. All of the above indicate the need for further research on fibrosis and the specific mechanisms of urethral stricture.

This study has the following strengths: We mainly focused on the European population, and its advantage is that it excludes the influence of population differences on the research results. The interference of reverse causal linkages and confounding factors is successfully reduced when the MR approach is implemented. In order to reduce the likelihood of weak tool bias, we have selected robust tools that come from sizable GWAS with a sizable enough sample size. This study strictly followed the MR process, minimizing sample overlap and ensuring the authenticity of the results.

This study does, however, have certain drawbacks. First off, even though we have performed numerous sensitivity studies, the idea that some genetic variations are pleiotropic cannot be totally ruled out. Second, the paucity of studies on the interplay between risk factors is a disadvantage of utilizing summary‐level data in two‐sample MR. Lastly, there may be limitations to generalizing the results of our study to other racial groups because the majority of GWAS participants are of European descent.

## 5. Conclusions

As a result, our study established the causal link between 37 immunological phenotypes and urethral stricture, which may offer some direction for the treatment of urethral stricture in the future. However, more work is required to fully understand the unique mechanisms of urethral stricture immune cells.

## Funding

This study was funded by the Health Commission of Shanxi Province (Grant 2024ZYYC051).

## Conflicts of Interest

The authors declare no conflicts of interest.

## Supporting Information

Additional supporting information can be found online in the Supporting Information section.

## Supporting information


**Supporting Information 1** S1 Appendix: Based on previous research, we have listed the STROBE‐MR checklist of Mendelian randomization studies to ensure the integrity of our processes.

**Supporting Information 2** S2 Appendix: The data source of immune phenotypes used in this study. These data were retrieved from the ieu open GWAS project.

**Supporting Information 3** S3 Appendix: The data source of urethral stricture was retrieved from FinnGen Consortium.

**Supporting Information 4** S4 Appendix: The R code used in this study.

**Supporting Information 5** S5 Appendix: The results of three Mendelian randomization methods of the impact of all immune phenotypes on urethral stricture. This study was mainly based on the method of inverse‐variance weighted.

**Supporting Information 6** S6 Appendix: The result of Cochran’s *Q* test, a *p*‐value greater than 0.05 is considered to have no heterogeneity.

**Supporting Information 7** S7 Appendix: The result of the MR‐Egger intercept test, the pleiotropy is considered when *p*  < 0.05.

**Supporting Information 8** S8 Appendix: The result of MR‐PRESSO, the two sets of results by the MR‐PRESSO test (CD27 on PB/PC (ID: ebi‐a‐GCST90001807), HLA DR + NK AC (ID: ebi‐a‐GCST90001648).

**Supporting Information 9** S9 Appendix: The result of reverse Mendelian analysis.

## Data Availability

The data that support the findings of this study are available in ieu OpenGWAS project at https://gwas.mrcieu.ac.uk/. These data were derived from the following resources available in the public domain: ‐ the ieu OpenGWAS project, https://gwas.mrcieu.ac.uk/.
