# Supplementary material for: Mendelian Randomization Reveals Genetic Associations Between Immune Traits and Urethral Stricture
Source: Mediators Inflamm. 2026 Feb 27;2026:3748167. doi: 10.1155/mi/3748167 (PMC12949340; doi:10.1155/mi/3748167)
Supplement: Supplementary file 5 — Supporting Information 5 S5 Appendix: The results of three Mendelian randomization methods of the impact of all immune phenotypes on urethral stricture. This study was mainly based on the method of inverse‐variance weighted. [file MI-2026-3748167-s009.pdf]

The results of Mendelian randomiza

id.exposur	id.outcome	outcome	exposure	method	nsnp	b	se
ebi-a-GCS	rEG9o0	outcome	|| id:ebi	MR Egger	24	0.0818847	0.0325557
ebi-a-GCS	rEG9o0	outcome	|| id:ebi	Weighted m	24	0.0815546	0.0418783
ebi-a-GCS	rEG9o0	outcome	|| id:ebi	Inverse va	24	0.0791568	0.0273966
ebi-a-GCS	kEUlr3	outcome	|| id:ebi	MR Egger	18	-0.042918	0.0338888
ebi-a-GCS	kEUlr3	outcome	|| id:ebi	Weighted m	18	-0.052742	0.0386027
ebi-a-GCS	kEUlr3	outcome	|| id:ebi	Inverse va	18	-0.058381	0.0283206
ebi-a-GCS	gTEekn	outcome	|| id:ebi	MR Egger	30	0.0823588	0.0400558
ebi-a-GCS	gTEekn	outcome	|| id:ebi	Weighted m	30	0.0829078	0.0480341
ebi-a-GCS	gTEekn	outcome	|| id:ebi	Inverse va	30	0.0821224	0.0319033
ebi-a-GCS	JAZAP3	outcome	|| id:ebi	MR Egger	28	-0.210831	0.0868145
ebi-a-GCS	JAZAP3	outcome	|| id:ebi	Weighted m	28	-0.208259	0.0764933
ebi-a-GCS	JAZAP3	outcome	|| id:ebi	Inverse va	28	-0.17962	0.0502128
ebi-a-GCS	7hjJJK	outcome	|| id:ebi	MR Egger	25	0.0483715	0.0353299
ebi-a-GCS	7hjJJK	outcome	|| id:ebi	Weighted m	25	0.0611381	0.0381154
ebi-a-GCS	7hjJJK	outcome	|| id:ebi	Inverse va	25	0.0569211	0.0289864
ebi-a-GCS	wzVjJF	outcome	|| id:ebi	MR Egger	33	-0.057143	0.0246706
ebi-a-GCS	wzVjJF	outcome	|| id:ebi	Weighted m	33	-0.055996	0.0298453
ebi-a-GCS	wzVjJF	outcome	|| id:ebi	Inverse va	33	-0.062675	0.021404
ebi-a-GCS	u6RNpD	outcome	|| id:ebi	MR Egger	20	0.1016987	0.0433267
ebi-a-GCS	u6RNpD	outcome	|| id:ebi	Weighted m	20	0.106587	0.0540496
ebi-a-GCS	u6RNpD	outcome	|| id:ebi	Inverse va	20	0.1059707	0.0363604
ebi-a-GCS	WMHVyx	outcome	|| id:ebi	MR Egger	21	-0.158417	0.1416234
ebi-a-GCS	WMHVyx	outcome	|| id:ebi	Weighted m	21	-0.193777	0.0874985
ebi-a-GCS	WMHVyx	outcome	|| id:ebi	Inverse va	21	-0.148896	0.063749
ebi-a-GCS	zERTed	outcome	|| id:ebi	MR Egger	21	0.1815461	0.0911573
ebi-a-GCS	zERTed	outcome	|| id:ebi	Weighted m	21	0.1522157	0.076951
ebi-a-GCS	zERTed	outcome	|| id:ebi	Inverse va	21	0.1192996	0.0525033
ebi-a-GCS	j0Qzqn	outcome	|| id:ebi	MR Egger	24	0.0258729	0.0426386
ebi-a-GCS	j0Qzqn	outcome	|| id:ebi	Weighted m	24	0.0102714	0.0493358
ebi-a-GCS	j0Qzqn	outcome	|| id:ebi	Inverse va	24	0.0735838	0.0303675
ebi-a-GCS	JTMO1m	outcome	|| id:ebi	MR Egger	18	0.1643014	0.0546793
ebi-a-GCS	JTMO1m	outcome	|| id:ebi	Weighted m	18	0.1659403	0.0489597
ebi-a-GCS	JTMO1m	outcome	|| id:ebi	Inverse va	18	0.1034756	0.0437187
ebi-a-GCS	CPrB87	outcome	|| id:ebi	MR Egger	24	-0.046792	0.0339616
ebi-a-GCS	CPrB87	outcome	|| id:ebi	Weighted m	24	-0.038549	0.036273
ebi-a-GCS	CPrB87	outcome	|| id:ebi	Inverse va	24	-0.04804	0.0231554
ebi-a-GCS	0KRj58	outcome	|| id:ebi	MR Egger	23	-0.164824	0.0706228
ebi-a-GCS	0KRj58	outcome	|| id:ebi	Weighted m	23	-0.125669	0.0731888
ebi-a-GCS	0KRj58	outcome	|| id:ebi	Inverse va	23	-0.14205	0.0484026
ebi-a-GCS	j1QyIq	outcome	|| id:ebi	MR Egger	22	-0.247916	0.1000476
ebi-a-GCS	j1QyIq	outcome	|| id:ebi	Weighted m	22	-0.163042	0.086836
ebi-a-GCS	j1QyIq	outcome	|| id:ebi	Inverse va	22	-0.1625	0.0575615
ebi-a-GCS	uj5xjD	outcome	|| id:ebi	MR Egger	17	-0.305493	0.0936661
ebi-a-GCS	uj5xjD	outcome	|| id:ebi	Weighted m	17	-0.16985	0.0702081
ebi-a-GCS	uj5xjD	outcome	|| id:ebi	Inverse va	17	-0.130187	0.0537108
ebi-a-GCS	Kbk9Da	outcome	|| id:ebi	MR Egger	14	-0.002264	0.152353
ebi-a-GCS	Kbk9Da	outcome	|| id:ebi	Weighted m	14	-0.093456	0.0976707
ebi-a-GCS	Kbk9Da	outcome	|| id:ebi	Inverse va	14	-0.164151	0.0691524
ebi-a-GCS	eWmswB	outcome	|| id:ebi	MR Egger	16	-0.167112	0.1718139

ebi-a-GCS1eWmswB	outcome	|| id:ebiWeighted m	16	-0.118582	0.0979686
ebi-a-GCS1eWmswB	outcome	|| id:ebiInverse va	16	-0.139395	0.0696696
ebi-a-GCS1k9XtQ7	outcome	|| id:ebiMR Egger	25	0.0771562	0.0305573
ebi-a-GCS1k9XtQ7	outcome	|| id:ebiWeighted m	25	0.060069	0.0320099
ebi-a-GCS1k9XtQ7	outcome	|| id:ebiInverse va	25	0.0719163	0.0233246
ebi-a-GCS1QIW6uJ	outcome	|| id:ebiMR Egger	20	-0.103715	0.0641861
ebi-a-GCS1QIW6uJ	outcome	|| id:ebiWeighted m	20	-0.102862	0.0730901
ebi-a-GCS1QIW6uJ	outcome	|| id:ebiInverse va	20	-0.127056	0.048106
ebi-a-GCS1ej3KfE	outcome	|| id:ebiMR Egger	31	0.0361358	0.0331386
ebi-a-GCS1ej3KfE	outcome	|| id:ebiWeighted m	31	0.0614645	0.0515019
ebi-a-GCS1ej3KfE	outcome	|| id:ebiInverse va	31	0.0584947	0.0254228
ebi-a-GCS1Z4VtJg	outcome	|| id:ebiMR Egger	19	0.0208021	0.0762294
ebi-a-GCS1Z4VtJg	outcome	|| id:ebiWeighted m	19	0.0854316	0.0779198
ebi-a-GCS1Z4VtJg	outcome	|| id:ebiInverse va	19	0.1510223	0.0627598
ebi-a-GCS1IJvBoP	outcome	|| id:ebiMR Egger	27	0.1035798	0.0564398
ebi-a-GCS1IJvBoP	outcome	|| id:ebiWeighted m	27	0.0994558	0.0532358
ebi-a-GCS1IJvBoP	outcome	|| id:ebiInverse va	27	0.1066487	0.0363662
ebi-a-GCS11HQIZv	outcome	|| id:ebiMR Egger	26	0.1011144	0.0588149
ebi-a-GCS11HQIZv	outcome	|| id:ebiWeighted m	26	0.066096	0.0500424
ebi-a-GCS11HQIZv	outcome	|| id:ebiInverse va	26	0.0680901	0.0343592
ebi-a-GCS1OYsxyZ	outcome	|| id:ebiMR Egger	36	0.0642386	0.0631693
ebi-a-GCS1OYsxyZ	outcome	|| id:ebiWeighted m	36	0.0929883	0.0586123
ebi-a-GCS1OYsxyZ	outcome	|| id:ebiInverse va	36	0.0840395	0.0388071
ebi-a-GCS1XrauL9	outcome	|| id:ebiMR Egger	20	-0.084223	0.0543784
ebi-a-GCS1XrauL9	outcome	|| id:ebiWeighted m	20	-0.102942	0.049446
ebi-a-GCS1XrauL9	outcome	|| id:ebiInverse va	20	-0.097125	0.0332391
ebi-a-GCS1A2ntFl	outcome	|| id:ebiMR Egger	3	0.2510079	1.0482578
ebi-a-GCS1A2ntFl	outcome	|| id:ebiWeighted m	3	-0.61366	0.2715999
ebi-a-GCS1A2ntFl	outcome	|| id:ebiInverse va	3	-0.494512	0.2181467
ebi-a-GCS1pukCPn	outcome	|| id:ebiMR Egger	22	0.1794604	0.1155199
ebi-a-GCS1pukCPn	outcome	|| id:ebiWeighted m	22	0.2681778	0.0762199
ebi-a-GCS1pukCPn	outcome	|| id:ebiInverse va	22	0.1648201	0.0583076
ebi-a-GCS1QLgMiA	outcome	|| id:ebiMR Egger	24	0.0634299	0.042809
ebi-a-GCS1QLgMiA	outcome	|| id:ebiWeighted m	24	0.048189	0.0402201
ebi-a-GCS1QLgMiA	outcome	|| id:ebiInverse va	24	0.0663292	0.0327144
ebi-a-GCS1Yy3Z8f	outcome	|| id:ebiMR Egger	19	-0.059431	0.0384181
ebi-a-GCS1Yy3Z8f	outcome	|| id:ebiWeighted m	19	-0.053039	0.0382011
ebi-a-GCS1Yy3Z8f	outcome	|| id:ebiInverse va	19	-0.070206	0.0320534
ebi-a-GCS145RZF8	outcome	|| id:ebiMR Egger	18	0.0810346	0.0342224
ebi-a-GCS145RZF8	outcome	|| id:ebiWeighted m	18	0.0292525	0.0404588
ebi-a-GCS145RZF8	outcome	|| id:ebiInverse va	18	0.0707223	0.02723
ebi-a-GCS1MUSzZJ	outcome	|| id:ebiMR Egger	20	-0.142897	0.04749
ebi-a-GCS1MUSzZJ	outcome	|| id:ebiWeighted m	20	-0.151321	0.0407229
ebi-a-GCS1MUSzZJ	outcome	|| id:ebiInverse va	20	-0.081396	0.03599
ebi-a-GCS11xHeAg	outcome	|| id:ebiMR Egger	21	0.2036148	0.0712271
ebi-a-GCS11xHeAg	outcome	|| id:ebiWeighted m	21	0.1206848	0.0493583
ebi-a-GCS11xHeAg	outcome	|| id:ebiInverse va	21	0.0808308	0.0386025
ebi-a-GCS1jZssKh	outcome	|| id:ebiMR Egger	14	0.1505373	0.1056253
ebi-a-GCS1jZssKh	outcome	|| id:ebiWeighted m	14	0.1755612	0.0686908
ebi-a-GCS1jZssKh	outcome	|| id:ebiInverse va	14	0.1650151	0.0520148
ebi-a-GCS1yBV7n8	outcome	|| id:ebiMR Egger	17	0.0249266	0.0689434

ebi-a-GCS1yBV7n8	outcome	|| id:ebiWeighted m	17	0.0762582	0.059726
ebi-a-GCS1yBV7n8	outcome	|| id:ebiInverse va	17	0.1021592	0.0393425
ebi-a-GCS1t14FBH	outcome	|| id:ebiMR Egger	22	-0.007872	0.0721799
ebi-a-GCS1t14FBH	outcome	|| id:ebiWeighted m	22	-0.063255	0.0649741
ebi-a-GCS1t14FBH	outcome	|| id:ebiInverse va	22	-0.090865	0.0447981
ebi-a-GCS1SIbrMz	outcome	|| id:ebiMR Egger	14	-0.149309	0.1376163
ebi-a-GCS1SIbrMz	outcome	|| id:ebiWeighted m	14	-0.153929	0.0913651
ebi-a-GCS1SIbrMz	outcome	|| id:ebiInverse va	14	-0.202347	0.0669366
ebi-a-GCS1TUotoV	outcome	|| id:ebiMR Egger	22	-0.123552	0.0518441
ebi-a-GCS1TUotoV	outcome	|| id:ebiWeighted m	22	-0.062369	0.0437051
ebi-a-GCS1TUotoV	outcome	|| id:ebiInverse va	22	-0.065276	0.0321051

ation analysis

pval	lo_ci	up_ci	or	or_lci95	or_uci95
0.02	0.0180755	0.1456939	1.09	1.02	1.16
0.05	-0.000527	0.1636361	1.08	1	1.18
0	0.0254595	0.132854	1.08	1.03	1.14
0.22	-0.10934	0.0235043	0.96	0.9	1.02
0.17	-0.128403	0.0229193	0.95	0.88	1.02
0.04	-0.113889	-0.002872	0.94	0.89	1
0.05	0.0038494	0.1608683	1.09	1	1.17
0.08	-0.011239	0.1770546	1.09	0.99	1.19
0.01	0.019592	0.1446528	1.09	1.02	1.16
0.02	-0.380987	-0.040675	0.81	0.68	0.96
0.01	-0.358186	-0.058332	0.81	0.7	0.94
0	-0.278038	-0.081203	0.84	0.76	0.92
0.18	-0.020875	0.1176181	1.05	0.98	1.12
0.11	-0.013568	0.1358444	1.06	0.99	1.15
0.05	0.0001079	0.1137344	1.06	1	1.12
0.03	-0.105498	-0.008789	0.94	0.9	0.99
0.06	-0.114493	0.0025011	0.95	0.89	1
0	-0.104627	-0.020724	0.94	0.9	0.98
0.03	0.0167784	0.1866189	1.11	1.02	1.21
0.05	0.0006498	0.2125242	1.11	1	1.24
0	0.0347043	0.1772371	1.11	1.04	1.19
0.28	-0.435999	0.1191645	0.85	0.65	1.13
0.03	-0.365274	-0.02228	0.82	0.69	0.98
0.02	-0.273844	-0.023948	0.86	0.76	0.98
0.06	0.0028777	0.3602145	1.2	1	1.43
0.05	0.0013917	0.3030397	1.16	1	1.35
0.02	0.0163932	0.222206	1.13	1.02	1.25
0.55	-0.057699	0.1094445	1.03	0.94	1.12
0.84	-0.086427	0.1069696	1.01	0.92	1.11
0.02	0.0140635	0.1331042	1.08	1.01	1.14
0.01	0.0571299	0.2714728	1.18	1.06	1.31
0	0.0699793	0.2619012	1.18	1.07	1.3
0.02	0.017787	0.1891642	1.11	1.02	1.21
0.18	-0.113356	0.0197731	0.95	0.89	1.02
0.29	-0.109644	0.0325461	0.96	0.9	1.03
0.04	-0.093425	-0.002656	0.95	0.91	1
0.03	-0.303245	-0.026403	0.85	0.74	0.97
0.09	-0.269119	0.0177812	0.88	0.76	1.02
0	-0.236919	-0.047181	0.87	0.79	0.95
0.02	-0.444009	-0.051823	0.78	0.64	0.95
0.06	-0.333241	0.0071562	0.85	0.72	1.01
0	-0.275321	-0.04968	0.85	0.76	0.95
0.01	-0.489078	-0.121907	0.74	0.61	0.89
0.02	-0.307457	-0.032242	0.84	0.74	0.97
0.02	-0.235461	-0.024914	0.88	0.79	0.98
0.99	-0.300876	0.2963475	1	0.74	1.34
0.34	-0.28489	0.097979	0.91	0.75	1.1
0.02	-0.29969	-0.028612	0.85	0.74	0.97
0.35	-0.503867	0.1696432	0.85	0.6	1.18

0.23	-0.3106	0.0734369	0.89	0.73	1.08
0.05	-0.275947	-0.002843	0.87	0.76	1
0.02	0.017264	0.1370485	1.08	1.02	1.15
0.06	-0.00267	0.1228084	1.06	1	1.13
0	0.0262001	0.1176325	1.07	1.03	1.12
0.12	-0.22952	0.0220893	0.9	0.79	1.02
0.16	-0.246119	0.0403944	0.9	0.78	1.04
0.01	-0.221343	-0.032768	0.88	0.8	0.97
0.28	-0.028816	0.1010874	1.04	0.97	1.11
0.23	-0.039479	0.1624082	1.06	0.96	1.18
0.02	0.008666	0.1083235	1.06	1.01	1.11
0.79	-0.128607	0.1702116	1.02	0.88	1.19
0.27	-0.067291	0.2381545	1.09	0.93	1.27
0.02	0.0280131	0.2740315	1.16	1.03	1.32
0.08	-0.007042	0.2142019	1.11	0.99	1.24
0.06	-0.004886	0.203798	1.1	1	1.23
0	0.0353709	0.1779264	1.11	1.04	1.19
0.1	-0.014163	0.2163917	1.11	0.99	1.24
0.19	-0.031987	0.1641791	1.07	0.97	1.18
0.05	0.000746	0.1354342	1.07	1	1.15
0.32	-0.059573	0.1880504	1.07	0.94	1.21
0.11	-0.021892	0.2078684	1.1	0.98	1.23
0.03	0.0079776	0.1601015	1.09	1.01	1.17
0.14	-0.190805	0.0223585	0.92	0.83	1.02
0.04	-0.199856	-0.006028	0.9	0.82	0.99
0	-0.162274	-0.031977	0.91	0.85	0.97
0.85	-1.803577	2.3055932	1.29	0.16	10.03
0.02	-1.145995	-0.081324	0.54	0.32	0.92
0.02	-0.92208	-0.066945	0.61	0.4	0.94
0.14	-0.046959	0.4058794	1.2	0.95	1.5
0	0.1187867	0.4175688	1.31	1.13	1.52
0	0.0505373	0.2791029	1.18	1.05	1.32
0.15	-0.020476	0.1473356	1.07	0.98	1.16
0.23	-0.030642	0.1270204	1.05	0.97	1.14
0.04	0.002209	0.1304494	1.07	1	1.14
0.14	-0.13473	0.0158685	0.94	0.87	1.02
0.17	-0.127913	0.0218357	0.95	0.88	1.02
0.03	-0.133031	-0.007381	0.93	0.88	0.99
0.03	0.0139587	0.1481106	1.08	1.01	1.16
0.47	-0.050047	0.1085517	1.03	0.95	1.11
0.01	0.0173515	0.124093	1.07	1.02	1.13
0.01	-0.235977	-0.049817	0.87	0.79	0.95
0	-0.231138	-0.071505	0.86	0.79	0.93
0.02	-0.151936	-0.010856	0.92	0.86	0.99
0.01	0.0640098	0.3432199	1.23	1.07	1.41
0.01	0.0239426	0.217427	1.13	1.02	1.24
0.04	0.00517	0.1564917	1.08	1.01	1.17
0.18	-0.056488	0.3575628	1.16	0.95	1.43
0.01	0.0409273	0.3101951	1.19	1.04	1.36
0	0.0630661	0.2669641	1.18	1.07	1.31
0.72	-0.110202	0.1600557	1.03	0.9	1.17

0.2	-0.040805	0.1933213	1.08	0.96	1.21
0.01	0.0250478	0.1792706	1.11	1.03	1.2
0.91	-0.149345	0.1336003	0.99	0.86	1.14
0.33	-0.190605	0.0640938	0.94	0.83	1.07
0.04	-0.178669	-0.003061	0.91	0.84	1
0.3	-0.419037	0.1204193	0.86	0.66	1.13
0.09	-0.333005	0.0251466	0.86	0.72	1.03
0	-0.333543	-0.071151	0.82	0.72	0.93
0.03	-0.225166	-0.021937	0.88	0.8	0.98
0.15	-0.148031	0.0232934	0.94	0.86	1.02
0.04	-0.128202	-0.00235	0.94	0.88	1
