# Supplementary material for: Mendelian Randomization Reveals Genetic Associations Between Immune Traits and Urethral Stricture
Source: Mediators Inflamm. 2026 Feb 27;2026:3748167. doi: 10.1155/mi/3748167 (PMC12949340; doi:10.1155/mi/3748167)
Supplement: Supplementary file 6 — Supporting Information 6 S6 Appendix: The result of Cochran’s Q test, a p‐value greater than 0.05 is considered to have no heterogeneity. [file MI-2026-3748167-s004.pdf]

The results of heterogeneity analysis

<table>
<tr><th></th><th>id.exposur</th><th>id.outcome</th><th>outcome</th><th>exposure</th><th>method</th><th>Q</th><th>Q_df</th><th>Q_pval</th></tr>
<tr><td>1</td><td>ebi-a-GCS1</td><td>5kzPMW</td><td>outcome</td><td>|| id:ebi</td><td>MR Egger</td><td>22.568882</td><td>22</td><td>0.4264046</td></tr>
<tr><td>2</td><td>ebi-a-GCS1</td><td>5kzPMW</td><td>outcome</td><td>|| id:ebi</td><td>Inverse va</td><td>22.595216</td><td>23</td><td>0.4846049</td></tr>
<tr><td>3</td><td>ebi-a-GCS1</td><td>ubC3o1</td><td>outcome</td><td>|| id:ebi</td><td>MR Egger</td><td>10.378365</td><td>16</td><td>0.8461366</td></tr>
<tr><td>4</td><td>ebi-a-GCS1</td><td>ubC3o1</td><td>outcome</td><td>|| id:ebi</td><td>Inverse va</td><td>11.068639</td><td>17</td><td>0.8529736</td></tr>
<tr><td>5</td><td>ebi-a-GCS1</td><td>504mRI</td><td>outcome</td><td>|| id:ebi</td><td>MR Egger</td><td>18.982567</td><td>28</td><td>0.8986729</td></tr>
<tr><td>6</td><td>ebi-a-GCS1</td><td>504mRI</td><td>outcome</td><td>|| id:ebi</td><td>Inverse va</td><td>18.982663</td><td>29</td><td>0.9217318</td></tr>
<tr><td>7</td><td>ebi-a-GCS1</td><td>tU9Dlr</td><td>outcome</td><td>|| id:ebi</td><td>MR Egger</td><td>20.204896</td><td>26</td><td>0.7817483</td></tr>
<tr><td>8</td><td>ebi-a-GCS1</td><td>tU9Dlr</td><td>outcome</td><td>|| id:ebi</td><td>Inverse va</td><td>20.399117</td><td>27</td><td>0.8135964</td></tr>
<tr><td>9</td><td>ebi-a-GCS1</td><td>SFK3Bv</td><td>outcome</td><td>|| id:ebi</td><td>MR Egger</td><td>28.381934</td><td>23</td><td>0.2016826</td></tr>
<tr><td>10</td><td>ebi-a-GCS1</td><td>SFK3Bv</td><td>outcome</td><td>|| id:ebi</td><td>Inverse va</td><td>28.620082</td><td>24</td><td>0.2347573</td></tr>
<tr><td>11</td><td>ebi-a-GCS1</td><td>XI3XkS</td><td>outcome</td><td>|| id:ebi</td><td>MR Egger</td><td>30.582787</td><td>31</td><td>0.4873703</td></tr>
<tr><td>12</td><td>ebi-a-GCS1</td><td>XI3XkS</td><td>outcome</td><td>|| id:ebi</td><td>Inverse va</td><td>30.786121</td><td>32</td><td>0.5278942</td></tr>
<tr><td>13</td><td>ebi-a-GCS1</td><td>pB6NAf</td><td>outcome</td><td>|| id:ebi</td><td>MR Egger</td><td>10.467637</td><td>18</td><td>0.9155688</td></tr>
<tr><td>14</td><td>ebi-a-GCS1</td><td>pB6NAf</td><td>outcome</td><td>|| id:ebi</td><td>Inverse va</td><td>10.500513</td><td>19</td><td>0.9395004</td></tr>
<tr><td>15</td><td>ebi-a-GCS1</td><td>xaew6O</td><td>outcome</td><td>|| id:ebi</td><td>MR Egger</td><td>13.627224</td><td>19</td><td>0.8049725</td></tr>
<tr><td>16</td><td>ebi-a-GCS1</td><td>xaew6O</td><td>outcome</td><td>|| id:ebi</td><td>Inverse va</td><td>13.632893</td><td>20</td><td>0.8486108</td></tr>
<tr><td>17</td><td>ebi-a-GCS1</td><td>c74CF7</td><td>outcome</td><td>|| id:ebi</td><td>MR Egger</td><td>18.112992</td><td>19</td><td>0.5149028</td></tr>
<tr><td>18</td><td>ebi-a-GCS1</td><td>c74CF7</td><td>outcome</td><td>|| id:ebi</td><td>Inverse va</td><td>18.810738</td><td>20</td><td>0.5341572</td></tr>
<tr><td>19</td><td>ebi-a-GCS1</td><td>RwHl3x</td><td>outcome</td><td>|| id:ebi</td><td>MR Egger</td><td>20.34128</td><td>22</td><td>0.5617013</td></tr>
<tr><td>20</td><td>ebi-a-GCS1</td><td>RwHl3x</td><td>outcome</td><td>|| id:ebi</td><td>Inverse va</td><td>22.882225</td><td>23</td><td>0.4676658</td></tr>
<tr><td>21</td><td>ebi-a-GCS1</td><td>KDnSdq</td><td>outcome</td><td>|| id:ebi</td><td>MR Egger</td><td>20.205395</td><td>16</td><td>0.2111119</td></tr>
<tr><td>22</td><td>ebi-a-GCS1</td><td>KDnSdq</td><td>outcome</td><td>|| id:ebi</td><td>Inverse va</td><td>23.879872</td><td>17</td><td>0.1227359</td></tr>
<tr><td>23</td><td>ebi-a-GCS1</td><td>w2A3RR</td><td>outcome</td><td>|| id:ebi</td><td>MR Egger</td><td>14.486902</td><td>22</td><td>0.8833015</td></tr>
<tr><td>24</td><td>ebi-a-GCS1</td><td>w2A3RR</td><td>outcome</td><td>|| id:ebi</td><td>Inverse va</td><td>14.489429</td><td>23</td><td>0.9119947</td></tr>
<tr><td>25</td><td>ebi-a-GCS1</td><td>rkTaoc</td><td>outcome</td><td>|| id:ebi</td><td>MR Egger</td><td>20.266112</td><td>21</td><td>0.504466</td></tr>
<tr><td>26</td><td>ebi-a-GCS1</td><td>rkTaoc</td><td>outcome</td><td>|| id:ebi</td><td>Inverse va</td><td>20.46222</td><td>22</td><td>0.554151</td></tr>
<tr><td>27</td><td>ebi-a-GCS1</td><td>kuAAc1</td><td>outcome</td><td>|| id:ebi</td><td>MR Egger</td><td>19.172284</td><td>20</td><td>0.5106541</td></tr>
<tr><td>28</td><td>ebi-a-GCS1</td><td>kuAAc1</td><td>outcome</td><td>|| id:ebi</td><td>Inverse va</td><td>20.261842</td><td>21</td><td>0.5047344</td></tr>
<tr><td>29</td><td>ebi-a-GCS1</td><td>RY2w8p</td><td>outcome</td><td>|| id:ebi</td><td>MR Egger</td><td>5.4969413</td><td>15</td><td>0.9870173</td></tr>
<tr><td>30</td><td>ebi-a-GCS1</td><td>RY2w8p</td><td>outcome</td><td>|| id:ebi</td><td>Inverse va</td><td>10.715946</td><td>16</td><td>0.8266697</td></tr>
<tr><td>31</td><td>ebi-a-GCS1</td><td>7anrp9</td><td>outcome</td><td>|| id:ebi</td><td>MR Egger</td><td>6.5422645</td><td>12</td><td>0.8863233</td></tr>
<tr><td>32</td><td>ebi-a-GCS1</td><td>7anrp9</td><td>outcome</td><td>|| id:ebi</td><td>Inverse va</td><td>7.964299</td><td>13</td><td>0.8459183</td></tr>
<tr><td>33</td><td>ebi-a-GCS1</td><td>EzSYIn</td><td>outcome</td><td>|| id:ebi</td><td>MR Egger</td><td>8.5391662</td><td>14</td><td>0.8593916</td></tr>
<tr><td>34</td><td>ebi-a-GCS1</td><td>EzSYIn</td><td>outcome</td><td>|| id:ebi</td><td>Inverse va</td><td>8.5703115</td><td>15</td><td>0.8988925</td></tr>
<tr><td>35</td><td>ebi-a-GCS1</td><td>87iDEs</td><td>outcome</td><td>|| id:ebi</td><td>MR Egger</td><td>24.692434</td><td>23</td><td>0.3663361</td></tr>
<tr><td>36</td><td>ebi-a-GCS1</td><td>87iDEs</td><td>outcome</td><td>|| id:ebi</td><td>Inverse va</td><td>24.772558</td><td>24</td><td>0.4182034</td></tr>
<tr><td>37</td><td>ebi-a-GCS1</td><td>l6hxkX</td><td>outcome</td><td>|| id:ebi</td><td>MR Egger</td><td>13.069442</td><td>18</td><td>0.7874312</td></tr>
<tr><td>38</td><td>ebi-a-GCS1</td><td>l6hxkX</td><td>outcome</td><td>|| id:ebi</td><td>Inverse va</td><td>13.371141</td><td>19</td><td>0.8190411</td></tr>
<tr><td>39</td><td>ebi-a-GCS1</td><td>lXlkzf</td><td>outcome</td><td>|| id:ebi</td><td>MR Egger</td><td>28.891325</td><td>29</td><td>0.4707368</td></tr>
<tr><td>40</td><td>ebi-a-GCS1</td><td>lXlkzf</td><td>outcome</td><td>|| id:ebi</td><td>Inverse va</td><td>29.997725</td><td>30</td><td>0.4657702</td></tr>
<tr><td>41</td><td>ebi-a-GCS1</td><td>VEQZ6d</td><td>outcome</td><td>|| id:ebi</td><td>MR Egger</td><td>18.908118</td><td>17</td><td>0.3338316</td></tr>
<tr><td>42</td><td>ebi-a-GCS1</td><td>VEQZ6d</td><td>outcome</td><td>|| id:ebi</td><td>Inverse va</td><td>25.76555</td><td>18</td><td>0.1052407</td></tr>
<tr><td>43</td><td>ebi-a-GCS1</td><td>bzqZEm</td><td>outcome</td><td>|| id:ebi</td><td>MR Egger</td><td>25.465537</td><td>25</td><td>0.4365414</td></tr>
<tr><td>44</td><td>ebi-a-GCS1</td><td>bzqZEm</td><td>outcome</td><td>|| id:ebi</td><td>Inverse va</td><td>25.470756</td><td>26</td><td>0.4924706</td></tr>
<tr><td>45</td><td>ebi-a-GCS1</td><td>86dE05</td><td>outcome</td><td>|| id:ebi</td><td>MR Egger</td><td>19.598568</td><td>24</td><td>0.7193605</td></tr>
<tr><td>46</td><td>ebi-a-GCS1</td><td>86dE05</td><td>outcome</td><td>|| id:ebi</td><td>Inverse va</td><td>20.077187</td><td>25</td><td>0.7427647</td></tr>
<tr><td>47</td><td>ebi-a-GCS1</td><td>qQAEtR</td><td>outcome</td><td>|| id:ebi</td><td>MR Egger</td><td>38.642211</td><td>34</td><td>0.267868</td></tr>
<tr><td>48</td><td>ebi-a-GCS1</td><td>qQAEtR</td><td>outcome</td><td>|| id:ebi</td><td>Inverse va</td><td>38.824289</td><td>35</td><td>0.3013508</td></tr>
<tr><td>49</td><td>ebi-a-GCS1</td><td>5IiwAa</td><td>outcome</td><td>|| id:ebi</td><td>MR Egger</td><td>16.156661</td><td>18</td><td>0.5816149</td></tr>
</table>

50	ebi-a-GCS15IiwAa	outcome	|| id:ebiInverse va	16.246534	19	0.6407709
51	ebi-a-GCS19Bib7T	outcome	|| id:ebiMR Egger	1.4737731	1	0.2247513
52	ebi-a-GCS19Bib7T	outcome	|| id:ebiInverse va	2.2637603	2	0.3224265
53	ebi-a-GCS1t1ADaC	outcome	|| id:ebiMR Egger	28.096162	20	0.1071425
54	ebi-a-GCS1t1ADaC	outcome	|| id:ebiInverse va	28.126953	21	0.1365874
55	ebi-a-GCS1NlmMK1	outcome	|| id:ebiMR Egger	29.706597	22	0.1257717
56	ebi-a-GCS1NlmMK1	outcome	|| id:ebiInverse va	29.722488	23	0.157535
57	ebi-a-GCS1m6j6My	outcome	|| id:ebiMR Egger	13.813461	17	0.6802595
58	ebi-a-GCS1m6j6My	outcome	|| id:ebiInverse va	14.072315	18	0.7243651
59	ebi-a-GCS1KS37J3	outcome	|| id:ebiMR Egger	17.359634	16	0.3627024
60	ebi-a-GCS1KS37J3	outcome	|| id:ebiInverse va	17.650742	17	0.4111878
61	ebi-a-GCS1GjCcv6	outcome	|| id:ebiMR Egger	22.252471	18	0.2209689
62	ebi-a-GCS1GjCcv6	outcome	|| id:ebiInverse va	26.479157	19	0.1173823
63	ebi-a-GCS174WYco	outcome	|| id:ebiMR Egger	17.666489	19	0.5448089
64	ebi-a-GCS174WYco	outcome	|| id:ebiInverse va	21.738511	20	0.3548667
65	ebi-a-GCS1T3FOfT	outcome	|| id:ebiMR Egger	6.0985132	12	0.9110348
66	ebi-a-GCS1T3FOfT	outcome	|| id:ebiInverse va	6.1233151	13	0.9415495
67	ebi-a-GCS1NPruNC	outcome	|| id:ebiMR Egger	7.4934301	15	0.9424852
68	ebi-a-GCS1NPruNC	outcome	|| id:ebiInverse va	9.3543359	16	0.898107
69	ebi-a-GCS1TdgyVV	outcome	|| id:ebiMR Egger	11.304034	20	0.9379536
70	ebi-a-GCS1TdgyVV	outcome	|| id:ebiInverse va	13.454414	21	0.8918756
71	ebi-a-GCS1rfOsTx	outcome	|| id:ebiMR Egger	5.6124471	12	0.9343456
72	ebi-a-GCS1rfOsTx	outcome	|| id:ebiInverse va	5.8070194	13	0.952895
73	ebi-a-GCS1W2Hc5W	outcome	|| id:ebiMR Egger	19.916543	20	0.4631611
74	ebi-a-GCS1W2Hc5W	outcome	|| id:ebiInverse va	21.914109	21	0.4044739
