# Supplementary material for: Mendelian Randomization Reveals Genetic Associations Between Immune Traits and Urethral Stricture
Source: Mediators Inflamm. 2026 Feb 27;2026:3748167. doi: 10.1155/mi/3748167 (PMC12949340; doi:10.1155/mi/3748167)
Supplement: Supplementary file 7 — Supporting Information 7 S7 Appendix: The result of the MR‐Egger intercept test, the pleiotropy is considered when p < 0.05. [file MI-2026-3748167-s005.pdf]

The results of pleiotropy analysis

	id.exposur	id.outcome	outcome	exposure	egger_inte	se	pval
1	ebi-a-GCS	15kzPMW	outcome	|| id:ebi	-0.002216	0.0138315	0.8741708
2	ebi-a-GCS	ubC3o1	outcome	|| id:ebi	-0.012282	0.0147834	0.4182973
3	ebi-a-GCS	504mRI	outcome	|| id:ebi	-0.000136	0.0139358	0.9922809
4	ebi-a-GCS	tU9Dlr	outcome	|| id:ebi	0.007087	0.0160809	0.6630688
5	ebi-a-GCS	SFK3Bv	outcome	|| id:ebi	0.0070858	0.0161295	0.6645399
6	ebi-a-GCS	XI3XkS	outcome	|| id:ebi	-0.004936	0.0109465	0.6551815
7	ebi-a-GCS	pB6NAf	outcome	|| id:ebi	0.0033224	0.0183236	0.8581443
8	ebi-a-GCS	xaew60	outcome	|| id:ebi	0.0019798	0.0262957	0.9407714
9	ebi-a-GCS	c74CF7	outcome	|| id:ebi	-0.017949	0.0214877	0.4139232
10	ebi-a-GCS	RwHl3x	outcome	|| id:ebi	0.027498	0.0172506	0.1251953
11	ebi-a-GCS	KDnSdq	outcome	|| id:ebi	-0.034543	0.0202507	0.1073814
12	ebi-a-GCS	w2A3RR	outcome	|| id:ebi	-0.000798	0.0158781	0.9603645
13	ebi-a-GCS	rkTaoc	outcome	|| id:ebi	0.0076466	0.017267	0.6624089
14	ebi-a-GCS	kuAAc1	outcome	|| id:ebi	0.0211885	0.020299	0.3090223
15	ebi-a-GCS	RY2w8p	outcome	|| id:ebi	0.0511251	0.022379	0.0373239
16	ebi-a-GCS	7anrp9	outcome	|| id:ebi	-0.037448	0.0314032	0.2561161
17	ebi-a-GCS	EzSYIn	outcome	|| id:ebi	0.0056269	0.031884	0.8624442
18	ebi-a-GCS	87iDEs	outcome	|| id:ebi	-0.004486	0.0164208	0.7871458
19	ebi-a-GCS	l6hxkX	outcome	|| id:ebi	-0.008335	0.0151751	0.5895692
20	ebi-a-GCS	lXlkzf	outcome	|| id:ebi	0.0130155	0.0123738	0.3015521
21	ebi-a-GCS	VEQZ6d	outcome	|| id:ebi	0.0460698	0.0185539	0.0237583
22	ebi-a-GCS	bzqZEm	outcome	|| id:ebi	0.0011323	0.0158187	0.9435085
23	ebi-a-GCS	86dE05	outcome	|| id:ebi	-0.012697	0.0183525	0.4956876
24	ebi-a-GCS	qQAEtR	outcome	|| id:ebi	0.0066058	0.016504	0.691472
25	ebi-a-GCS	5IiwAa	outcome	|| id:ebi	-0.005963	0.0198915	0.7677727
26	ebi-a-GCS	9Bib7T	outcome	|| id:ebi	-0.11362	0.1551881	0.5976735
27	ebi-a-GCS	t1ADaC	outcome	|| id:ebi	-0.003888	0.0262644	0.8837876
28	ebi-a-GCS	NlmMK1	outcome	|| id:ebi	0.0018598	0.0171444	0.9145987
29	ebi-a-GCS	m6j6My	outcome	|| id:ebi	-0.007994	0.0157115	0.6174479
30	ebi-a-GCS	KS37J3	outcome	|| id:ebi	-0.010015	0.0193343	0.6115576
31	ebi-a-GCS	GjCcv6	outcome	|| id:ebi	0.0360441	0.0194934	0.0809446
32	ebi-a-GCS	74WYco	outcome	|| id:ebi	-0.049166	0.0243646	0.0579453
33	ebi-a-GCS	T3FOfT	outcome	|| id:ebi	0.0052452	0.033306	0.8774806
34	ebi-a-GCS	NPruNC	outcome	|| id:ebi	0.0375729	0.0275431	0.1926399
35	ebi-a-GCS	TdgyVV	outcome	|| id:ebi	-0.027277	0.0186011	0.1580825
36	ebi-a-GCS	rfOsTx	outcome	|| id:ebi	-0.014033	0.0318124	0.6669806
37	ebi-a-GCS	W2Hc5W	outcome	|| id:ebi	0.0266467	0.0188535	0.1729278
